# Predictors of mental distress among undergraduate health science students of Hawassa University, College of Medicine and Health Sciences, Hawassa, SNNPR, Ethiopia: a cross-sectional study

**DOI:** 10.1186/s12991-020-0258-y

**Published:** 2020-02-03

**Authors:** Asres Bedaso, Bereket Duko, Tebikew Yeneabat

**Affiliations:** 1 Hawassa University, College of Medicine and Health Sciences, School of Nursing, P.O. Box 1560, Hawassa, Ethiopia; 2 College of Health Sciences, Department of Midwifery, Debre Markos University, Debre Markos, Amhara, Ethiopia

**Keywords:** Mental distress, Predictors, Health science students

## Abstract

**Introduction:**

Mental distress is a mental health problem which includes anxiety, depression and somatic symptoms. Mental health problems affect society as a whole and no group is immune to mental disorders; however, students have significantly high level of mental distress than their community peers.

**Objective:**

The aim of the study is to assess magnitude of mental distress and its predictors among undergraduate health science students of Hawassa University, College of Medicine and Health Sciences, SNNPR, Ethiopia.

**Methods:**

Institution-based cross-sectional study was conducted among 311 students. Simple random sampling technique was used to select the study participants. Data were collected using pre-tested and structured self-administered questionnaire. Mental distress among students was assessed using SRQ-20, which is validated in Ethiopia. Bivariate and multivariate logistic regression model was fitted to identify predictors of mental distress among students. An adjusted odds ratio with 95% confidence interval was computed to determine the level of significance with *P*-value less than 0.05.

**Result:**

A total of 309 study participants were interviewed with a response rate of 99.34%. Among the total respondents 105 (34%) of them were found to have mental distress. In multiple logistic regression analysis, poor social support (AOR = 5.28; 95% CI (2.176–12.84) and current substances use (AOR = 12.83, 95% CI (7.13–23.13), were significant predictors of mental distress among respondents.

**Conclusion and recommendations:**

The overall magnitude of mental distress among students was found to be high. Therefore, it is recommended that mental distress needs due attention and remedial action from policy-makers, college officials, non-governmental organizations, parents, students and other concerned bodies.

## Introduction

Mental distress is a mental health problem which includes anxiety, depression and somatic symptoms [1]. Currently mental distress is a major public mental health problem and a leading cause of disability worldwide, accounting for one-third of disability adjusted life years [2]. In Africa mental illness particularly, mental distress is an important public health challenge that is under-recognized as a public problem [3].

Studies conducted among undergraduate students in Canada showed that 30% of students had mental distress which was significantly higher than adults in the general population of Canada [4]. Also more than half of students in USA [5] and 53% of students in Australia had mental distress [6].

A study conducted in South Africa, Malaysia, Kenya and Ethiopia revealed the prevalence of common mental disorders among college students is 27% [3], 41.9% [7], 10.8% [8] and 21.6% [9], respectively.

In Ethiopia, mental distress accounts for 11% of the total burden of diseases [10]. Although mental health problems affect all society and no group is immune to psychiatric disorders, students have significantly high level of psychological distress than their community peers [11].

This is due to the fact that university students face multiple stressors such as academic load, constant pressure to succeed, competition with peers, financial problems, peer pressure, teacher or parental pressure as well as concerns about the future [12]. This can have negative effects on student’s ability to study and academic outcomes [13]. If not properly managed, such situation of stress may later lead students to develop mental distress [14].

Another study conducted in Ethiopia revealed that 32.6% of medical students experienced mental distress [15]. However, since this study was done only on medical students and with small sample size, it may not represent all university students in Ethiopia.

A cross-sectional study conducted in University of Gondar the prevalence of mental distress was 40.9% [16]. In a study conducted in Haramaya University, the prevalence of mental distress was found 19.3% [17] and in Hawassa University among medical students the prevalence was 30% [18]. Due to the fact that there were few published literatures regarding the issue in our country, especially among health science students, this study was conducted with the aim of serving as a baseline for further study and to recommend the concerned bodies to intervene on this target group of population.

## Method

### Study design and period

Institution-based cross-sectional study design was conducted to assess the magnitude of mental distress and its predictors among undergraduate health science students of Hawassa University, College of Medicine and Health Sciences, Hawassa City, SNNPR, Ethiopia, from January 29 to February 14/2017 GC which was exam-free period.

This study was conducted in Hawassa University College of Medicine and Health Sciences, undergraduate health science students in Hawassa located 275 km from Addis Ababa. The college is one of the eight colleges under Hawassa University. The college is established to teach medical, nursing, midwifery, public, environmental health, psychiatric nursing, optometry, anesthesiology, laboratory and radiology students. In the academic year 2016/2017 the college had enrolled a total of 1570 health science students.

### Sample size determination and sampling procedures

The participants of this study were undergraduate health science students enrolled in the year 2016/2017 and attending classes in regular program under College of Medicine and Health Science.

Students were stratified based on the year of study. Since there was no study done among health science students, sample size was calculated by assuming the prevalence of mental distress 50%, 95% confidence interval and 5% margin of error. Considering the total population (*N* = 1170 health science students) correction formula was used and adding 10% for non-response the final sample size was 309. Simple random sampling technique was used to select the 309 study subjects to be included in the study.

### Data collection instrument

Data were collected using self-administered technique. The English version of the first part of the tool contains socio-demographic characteristics of students. The second part of the questionnaire asks about behavioral factors, which includes history of substance use (alcohol use, chat chewing and cigarette smoking) of students and the third part of the questionnaire assesses about social support of students using 3-item Oslo Social Support Scale. The fourth part of the questionnaire is self-reporting questionnaire (referred to as SRQ-20). Self-reporting questionnaire was used to estimate the prevalence of mental distress among health science students. This self-reporting questionnaire is a standardized questionnaire having 20-item questions, originally developed by World Health Organization (WHO) designed to indicate mental distress. The tool is adopted from WHO and was validated in low- and middle-income countries including Ethiopia. Students who were found to have 8 or more symptoms of the 20-item self-reporting questionnaires (SRQ-20) in the last 4 weeks were considered as having mental distress. The cut-off point was used based on the reports from the validation study of SRQ-20 that gave the highest sensitivity and specificity which corresponds to a cut-off point of 7 [19].

Social support of the study participants was assessed using 12-item Multidimensional Scale of Perceived Social Support Tool [20]. The items are divided in to factor groups relating to the source of social support, namely family, friends and significant others. Each item is scored from one (very strongly disagree) to 7 (very strongly agree). The total sum of all the 12 items possibly ranges from 12 to 84. A score 69–84 considered as high level of social support, whereas a score of 49–68 and 12–48 were considered as moderate and low level of social support, respectively. The reliability of the tool was checked using Cronbach's alpha reliability test with a score of 0.82 (95% CI 0.801–0.837).

Pre-test was conducted on 5% of sample size in Dilla University to ensure the reliability of the tool with Cronbach's *α* = 0.79. The collected data were reviewed and checked for completeness by group members on each day.

### Data processing and analysis

After the collected data were reviewed and checked for completeness before data entry, the incomplete data were discarded (questionnaires were incomplete). Data were cleaned, edited, coded and entered into Epi-info version 3.5.1 and exported in to SPSS version 20 software for analysis and we used both descriptive and analytical statistical procedures. Descriptive statistics like percentage mean and standard deviation was used for the presentation of demographic data and magnitude of mental distress. Tables were also used for data presentation. Each independent variable against the dependent variable was tested for having statistical significant association using binary logistic regression. Multiple logistic regression analysis was done for those variables with P-value less than or equal to 0.2 during binary logistic regression analysis.

### Ethical consideration

Ethical clearance was obtained from the institutional review board (IRB) of Hawassa University. Ethical review board approved the methods of data collection and forms of consent. Each questionnaire was prepared with the written consent attached with it to offer the consent for the respondents verbally and participants willing to be interviewed had signed and finally the obtained consents were kept with the questionnaire. The respondents were informed that their inclusion in the study is voluntary and they are free to withdraw from the study if they are not willing to participate. Anonymity was maintained to ensure confidentiality.

## Result

### Socio-demographic characteristics of study participants

A total of 309 other health science students were assessed, of which 193 (62.5%) were males and 115 (37.2%) of them were second year students. Respondents’ age ranged from 18 to 35 years, with a mean (± SD) of 22.2 (± 2.48) years. The higher percentages of the respondents were from urban background 208 (67.3%). Majority of the participants 130 (42.1%) were followers of Orthodox religion (Table 1).

**Table 1:** Distribution of socio-demographic characteristics among undergraduate health science students of Hawassa University College of Medicine and Health Sciences, SNNPRS, Ethiopia, May 2017 GC

Characteristics	Category	Frequency	Percent (%)
Sex	Male	193	29
	Female	116	71
Age	18–20	103	36.8
	21–23	115	38.9
	24–26	78	16.2
	> 27	13	8.1
Residence	Urban	208	67.3
	Rural	101	32.7
Religion	Protestant	92	29.8
	Orthodox	130	42.1
	Muslim	59	19.1
	Catholic	23	7.4
	Other	5	1.6
Year of study	1st year	56	18.1
	2nd year	115	37.2
	3rd year	82	26.5
	4th year	56	18.1
Ethnicity	Amhara	89	28.8
	Oromia	86	27.8
	Tigray	29	9.4
	Wolaita	25	8.1
	Gurage	21	6.8
	Other	59	19.1
Marital status	Single	273	88.3
	Married	20	6.5
	Divorced	5	1.6
	Other	11	3.6
Monthly income	< 735ETB per month	217	70.2
	735-1176ETB per month	44	14.2
	> 1176ETB per month	48	15.5

### Social support of respondents

In the assessment of social support using 3-item Oslo Social Support Scale (OSS-3), out of 309 students, more than half of the respondents 231 (74.7%) had strong social support and the rest 78 (25.2%) had poor social support.

### Magnitude of mental distress

Magnitude of mental distress using SRQ-20, with a cut-off point of 7 and above was 34% (95% CI 32.3, 35.7) (105 students out of 309 students). The distribution of SRQ-20 showed a mean value of 5.27 (± 4.31) ranging from 0 to 20.

### Factors associated with mental distress

During multiple logistic regression analysis, poor social support (AOR = 5.28; 95% CI (2.176–12.84), and current substances use (AOR = 12.84, 95% CI **(**7.13–23.13**)**, were significant predictors of mental distress among students (Table 2).

**Table 2:** Factors associated with mental distress among undergraduate health science students of Hawassa University College of Medicine and Health Sciences, SNNPRS, Ethiopia, June 2017 GC

Characteristics	Mental distress				
	Yes	No	COR (95% CI)	AOR (95% CI)	*P* value
Age					
18–20	71	32	0.20 (0.057,0.669)	0.42 (0.83,2.154)	0.299
21–23	80	35	0.194 (0.56,0.674)	0.28 (0.58,1.338)	0.111
24–26	49	29	0.263 (0.74, 0.931)	0.49 (0.11,2.385)	0.378
> 27	4	9	1	1	1
Year of study					
1st year	40	16	1	1	1
2nd year	79	36	0.87 (0.565,2.3		
3rd year	52	30	0.69 (0.693,3)		
4th year	33	23	1.742 (0.793,3.83)		
Marital status					
Married	11	9	3.68 (0.629,21.559)	0.43 (0.12,1.58)	0.204
Divorced	4	1	2.33 (0.492,1098)	0.16 (0.88,3.039)	0.222
Single	180	93	1.125 (0.078,16.3)	0.589 (0.054,6.46)	0.665
Other	9	2	1	1	1
Current substance use					
Yes	44	81	12.27 (6.97,21.58)	*12.84 (7.13,23.13)*	*0.001*^a^
No	160	24	1	1	1
Social support					
Poor	71	7	7.47 (3.29,16.95)	*5.28 (2.18, 12.84)*	*0.001*^a^
Strong	133	98	1	1	1

*AOR* adjusted odds ratio, *COR* crudes odds ratio, *CI* confidence interval

^a^Significant association (*p* < 0.01)

### Discussion

The magnitude of mental distress among students in this study was found to be 34%. The finding in the current study is lower compared to studies in USA (57%) [5], Australia (53%) [6], Brazil (44.7%) [21], Gondar (40.9) [16]. The variation might be due to the socio-cultural, environmental factors. Also time variation, the improvement of infrastructure and a service option provided by school authorities from time to time could be the reason for the variation.

However, the magnitude in the current study was higher when compared with studies in France (25.7%) [13], Norway (22.9%) [22] and Iceland (22.5%) [23] and Australia (19.2%). This could be due to the different instrument used in other studies or it could be a real difference. However, nearly similar prevalence was reported in Ethiopia revealed that 32.6% of medical students experienced mental distress [15].

Substance use was found to be a strong predictor of mental distress (AOR = 12.84, 95% CI (7.13–23.13), the odds of students who ever use substance were 12.8 times more likely to have mental distress as compared to the odds of students who never use substance. This finding is in line with other studies in Ethiopia [9] and Sao Paulo, Southeastern Brazil [24]. This might be because, substance use leads to inefficiency in life function, impaired relationship and sleep deprivation. Furthermore, substance use is associated with increased absenteeism from class and poor academic performance which can further lead to mental distress in students.

In this study, social support was also found to be another determinant factor for mental distress in students (AOR = 5.28; 95% CI (2.176–12.835). Having low level of social support from significant others were positively associated with mental distress. In this study, the odds of students with low social support were 5.3 times more likely to have mental distress as compared to those students with good social support. This finding is also in line with study conducted in Norwegian [22]. This could be due to its effect on hypothalamic pituitary adrenocortical (HPA) system in reducing genetic and environmental vulnerabilities. Furthermore, it is also important for maintaining good physical and mental health [25].

### Conclusion and recommendation

The prevalence of mental distress among students was found to be high. Having low social support and ever use of substance were strong predictors of mental distress.

Therefore, it is recommended that mental distress needs due attention and remedial action from policy-makers, college officials, non-governmental organizations, parents, students and other concerned bodies. Programs aimed at preventing mental distress need to address these identified factors of mental distress against students.

## Limitation

The cross-sectional nature of the study design does not confirm definitive cause-and-effect relationship. Also, the study may be prone to reporting bias since the data were collected based on self-reported information.

## Data Availability

All analyzed and generated data are included in this article and its supporting document.
